# MXene Heterostructures as Perspective Materials for Gas Sensing Applications

**DOI:** 10.3390/s22030972

**Published:** 2022-01-27

**Authors:** Svitlana Nahirniak, Bilge Saruhan

**Affiliations:** German Aerospace Center, Department of High-Temperature and Functional Coatings, Institute of Materials Research, Linder Hoehe, 51147 Cologne, Germany; Bilge.Saruhan@dlr.de

**Keywords:** two-dimensional structures, MXene, heterostructures, gas sensor, metal oxides, non-fluorine synthesis routes

## Abstract

This paper provides a summary of the recent developments with promising 2D MXene-related materials and gives an outlook for further research on gas sensor applications. The current synthesis routes that are provided in the literature are summarized, and the main properties of MXene compounds have been highlighted. Particular attention has been paid to safe and non-hazardous synthesis approaches for MXene production as 2D materials. The work so far on sensing properties of pure MXenes and MXene-based heterostructures has been considered. Significant improvement of the MXenes sensing performances not only relies on 2D production but also on the formation of MXene heterostructures with other 2D materials, such as graphene, and with metal oxides layers. Despite the limited number of research papers published in this area, recommendations on new strategies to advance MXene heterostructures and composites for gas sensing applications can be driven.

## 1. Introduction

Gas sensing devices have currently become a part of our daily lives, and their application areas continue to increase and demand the development of reliable, highly sensitive and selective gas sensing materials as well as technologically developed sensor designs. Among different types of gas sensing systems, chemoresistive gas sensors attract the most interest due to the set of their functional characteristics, such as superior sensing performance, low cost, smooth operation and possible miniaturization [1,2]. Since the first chemoresistive metal oxide gas sensor based on ZnO film was developed [3], a vast amount of work has been conducted to improve their working characteristics, including sensitivity, selectivity and stability. On the other hand, the commercialized gas sensors still operate at elevated temperatures, which leads to high-power consumption, reduced sensor stability, limited selectivity, decreased lifetime and limited application areas [2,4,5]. Therefore, the development and investigation of new materials is a crucial issue for obtaining new sensing systems with improved performance and excellent room temperature sensing ability.

**Figure 1:**
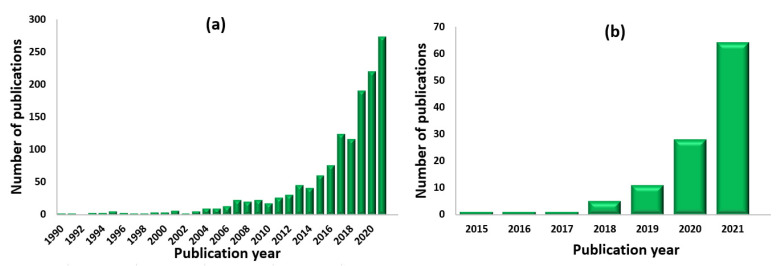
The number of publications in the area of 2D-materials-based gas sensors: (**a**) from 1990 to 2021, keyword for search: 2D gas sensors; (**b**) from 2015 to 2021, keyword for search: MXene gas sensors. Internet search of the Scopus on 17 January 2022.

Two-dimensional structures, such as graphene, transition metal dichalcogenides, transition carbides and hybrid 2D compounds, have attracted big interest for various gas sensing applications due to their large surface area, controlled surface chemistry and capability of sensing detection at room temperature [6,7]. The great progress in the development of gas sensors, especially based on 2D materials, can be seen in the increasing number of scientific papers published in this area (see Figure 1a). Sensing performance of 2D structures depends on several factors, including their thickness (which may vary from a few nanometers to a few centimeters), composition, number and quality of the atomistic layers [8]. The structural parameters of the 2D materials can be adjusted by optimizing the synthesis route, among which the most popular ones are exfoliation techniques [9], chemical and physical vapor deposition [10,11] and wet-chemical approaches [12]. In turn, the composition of 2D nanosheets or nanoflakes can be controlled due to the modification, which allows the significant optimization of their functional properties [8].

To date, several factors have been established that have a significant impact on the sensor performance, including structural defects, surface-terminated groups (for instance, oxygen-containing), surface functions and dopants [13]. The sensitivity of the nanostructured gas sensor system may be significantly improved due to the surface functionalization; however, selectivity is still not satisfactory for most of the resistive gas sensors [13]. So far, several strategies have been proposed for the enhancement of sensor selectivity, including surface functionalization with a second phase using noble metals or metal oxides [14,15,16], fabrication of composites or heterostructures [17,18], UV-illumination [19] or designing of multi-array sensor systems [20]. For instance, it is known that the formation of hybrid materials results in a combination of properties that are not available in single materials [8]. However, it shall be noted that the sensing mechanism of such hybrid materials becomes more complicated due to the interaction of gas molecules with diverse material components, as well as due to the interface interaction between different materials [13].

Among the big family of two-dimensional materials, MXenes and MXene-based structures recently gained particular attention for gas sensor-related applications due to their large surface-to-volume ratio, superior surface conductivity and surface-terminated functionality [21]. Until now, MXene demonstrated great perspectives for the development of energy storage devices. Since the first investigation of Mxenes’ gas performance in 2015, the number of publications in this area increases each year (see Figure 1b) by multiplying; however, the investigation of their sensing performance is still in the preliminary stage [22]. For instance, the selectivity of these systems still remains an issue, so further research efforts shall be applied to enhance and optimize their gas sensor performance, for example, by surface functionalization, coating or fabrication of hybrid materials [8].

Therefore, this review acts as a stepping stone to further research studies on MXene structures applied for gas sensing by summarizing achievements and indicating weaknesses of the past works and the up-to-date research. In this work, we discuss both theoretical (e.g., theoretical simulation using density functional theory) and experimental studies on gas sensing by MXenes that have been reported so far. Moreover, the strategies that are employed for the improvement of their sensor performance, such as decoration with metallic nanoparticles and creation of hybrid composite structures, have also been reviewed. The approaches to obtain pure MXenes and MXene-based composites and heterostructures with an emphasis on ecologically safe synthesis methods have been provided. Based on the available studies, we aim to show that the resulting sensing characteristics may be adjusted by modifying the final morphology by means of various synthesis techniques, process parameters as well as the exfoliation method.

## 2. Two-Dimensional Materials for Sensing Applications

The most important quality indicators of gas sensor performance (sensitivity, selectivity, response and recovery time, detection limit, etc.) strongly depend on characteristics of the sensing material. For instance, the large surface area of sensing material contributes to the interaction between the material’s surface and the target gas molecules, while the presence of active surface sites provides effective and selective adsorption of gas molecules [23]. From this point of view, two-dimensional nanostructures, unique material properties of which are naturally different from the bulk structures and deserve special attention for the development of high-performance gas sensors. Besides providing a large surface area and more active sites, 2D materials are characterized by a number of additional advantages, including a tunable electronic structure, facile surface functionalization, the possibility of 3D architectures assembling, excellent flexibility, good combability with device integration and outstanding mechanical robustness [8,24]. Due to the usage of 2D structures, the “4S” sensor performance characteristics (sensitivity, selectivity, stability and speed—response/recovery time) have significantly improved [8].

For the gas sensing applications, (i) metal oxide nanosheets [25,26,27,28], (ii) graphene-based structures [29,30,31] and (iii) dichalcogenides [32,33,34] are the most studied 2D materials.

(i) Metal oxide-based structures are characterized by high sensitivity to gas molecules and good stability. Besides their cheap and easy production in diverse and various nanostructured morphologies can be achieved [5,35]. The principle for operating metal oxide sensors is based on the sensing surface layer’s conductivity changes depending on the presence of gas in the environment. In general, the sensing mechanism includes several stages: adsorption of oxygen species on the semiconductor’s surface; electron transfer between semiconductor and oxygen; adsorption of the detected gas; chemical reaction; transfer of electron to the semiconductor; products desorption. In this case, the nanostructure and morphology of sensing materials has a great influence on the sensor performance. For example, a porous structure leads to an increase in the surface-to-volume ratio, and a large specific area provides more active sites for the adsorption of gas molecules [5,8].
(ii) Graphene-based materials are promising candidates for the detection of gaseous molecules due to their high electrical conductivity, extremely high specific surface area and high charge carrier mobility [8,36,37]. The sensing principle of the graphene and graphene-derived structures is based on the direct charge transfer mechanism due to the adsorption/desorption of gas molecules, which leads to a change in the local charge carrier concentration [38]. Depending on the gas nature (electron donor or acceptor), an increase or decrease in electrical conductivity occurs.
(iii) Two-dimensional layered structures of transition metal dichalcogenides, including MoS_2_, MoSe_2_ and WS_2_, are also characterized by good semiconducting properties, high surface area and excellent surface sensitivity, resulting in their widespread application for gas detection [33,39,40]. The sensing mechanism of metal dichalcogenides is similar to graphene-based materials and based on charge transfer between the surface and adsorbed molecules [41].

Besides the good sensing performance, 2D materials can provide a good basis for the development of gas sensors that are capable of low- or room-temperature operation [7]. For instance, Zhang and Yin demonstrated the high ethanol-sensing properties of SnO_2_ nanosheets at a low operating temperature of 165 °C. Regarding the good sensor performance, the authors explained that the mesoporous texture of the obtained nanosheets, in combination with small grain sized and surface defects, results in high response, fast response/recovery and good selectivity [42].

In contrast to the metal oxide-based gas sensors, which are able to operate at low temperatures, graphene-based materials and transition metal dichalcogenides offer the possibility to develop room-temperature sensors. Thus, the formation of graphene/CNT hybrid films or the decoration of graphene with Pd nanoparticles allow obtaining high-performance NO_2_ sensors’ operation at room temperatures, opening the possibility for the development of low-power sensor devices [43,44]. Sensors based on WS_2_ nanoflakes or WS_2_ and MoS_2_ thin films show great perspectives for the fabrication of room-temperature ammonia sensors with high sensitivity and selectivity [40,45]. MXene is another class of 2D compounds that are ideal candidates for the development of high-performance sensor devices, especially for the low- and room-temperature operation, due to their exceptional electronic, physical, chemical and mechanical properties, including large specific surface area, very narrow and tunable bandgap, fast electron transfer ability and adjustable surface chemistry [24,46,47,48]. MXenes are characterized by 2D layered graphene-like morphology, but in contrast to the other 2D materials, they exhibit higher responses with a high signal-to-noise ratio, providing the relative intensity of the gas signal over the noise intensity due to the strong binding of functional groups with analytes [6,48]. The near-free electron states of the MXene structures are located around the Fermi level, allowing fast charge-carrier transport through the electron transport channels [49]. Thus, in general, the superior sensing properties of MXenes are attributed to the numerous surface functional groups, which form strong bonds with analyte gases, and their metallic conductivity, which allows fast electron transfer and mobility [48]. For instance, the selectivity of a sensor based on MXene structures depends strongly on several factors, including the interaction between surface and gas molecules; MXene compositions and charge states; MXene flakes orientation [50]. Moreover, the controllable surface terminations provide great prospects for the modification of MXene’s structures, resulting in the improvement of their properties and sensing performance [6].

## 3. MXENES—Novel Two-Dimensional Compounds

**Figure 2:**
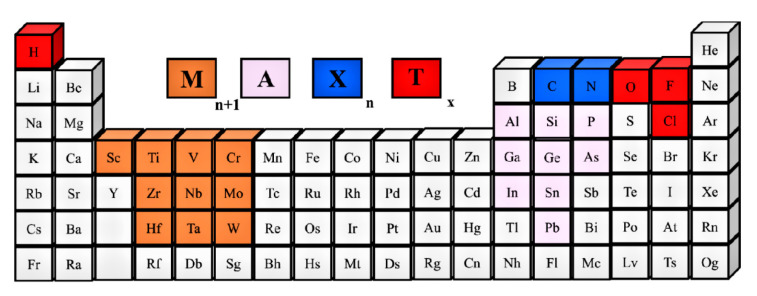
Periodic table showing MAX composition. Reprinted with permission from [52]. Copyright © 2021, American Chemical Society, CC-BY-NC-ND 4.0 license.

The so-called MXene compounds were discovered by Gogotsi et al. and first described in 2011 in the journal Advanced Materials [51]. MXene belongs to the group of transition metal carbides and nitrides and is synthesized from M*_n_*_+1_AX*_n_* phases (*n* = 1–3) (see Figure 2) by selectively etching the intermediate A layer using fluoride-ion-containing solutions [51,52]. MXene compounds are characterized by the numerous oxygen, hydroxyl and fluorine functional groups, which determine the material’s properties [53].

**Figure 3:**
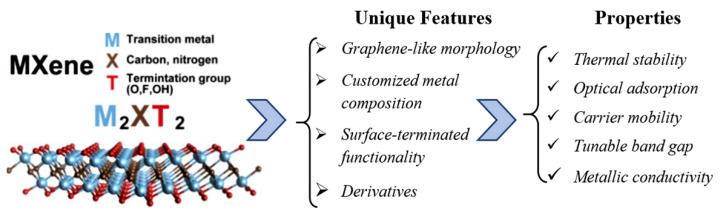
Schematic illustration of features and properties of MXenes. Reprinted with permission from [56]. Copyright © 1969, Elsevier.

MXene compounds’ most remarkable features include: graphene-like morphology, metallic conductivity, large surface area, mechanical flexibility and strong hydrophilic surface-terminated functionality [21]. The inherent structure and chemical composition of MXenes bring them unique physical and chemical characteristics (see Figure 3), which can be tailored to diverse applications [54]. The electronic properties of the exfoliated MXene layers are a function of the surface terminations. Thus, excellent electrical conductivity and semiconducting behavior due to the surface terminations (hydroxyl- or oxygen-terminated surfaces are responsible for the metallic conductivity, while termination with OH and F groups leads to the semiconducting character of the band structure) [51] in combination with the large surface area, good adsorption properties, high surface reactivity and large number of active sites make MXenes the ideal candidates for gas-sensing applications [21,23,55].

### 3.1. Synthesis Approaches for the MXenes Production

**Figure 4:**
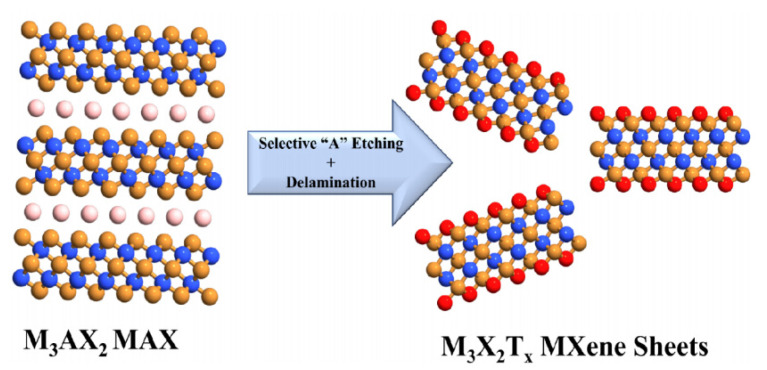
Schematic illustration of MXene synthesis. Reprinted with permission from [52]. Copyright © 2021, American Chemical Society, CC-BY-NC-ND 4.0 license.

As mentioned above, MXenes production is achieved by the selective extraction and exfoliation from MAX phases due to the higher chemical activity of the M–A bond compared to the metallic M–X bonds [54]. Figure 4 presents the schematic illustration of the MXenes synthesis. In MAX and MXene (M_2_XT_2_) formulas, M corresponds to the transition metals (for instance, Ti, Zr, V, Nb, Ta, etc.), X represents C or N and A refers to the elements from the 13 or 14 groups of the Periodic Table (such as Al, Si, P, Ge, Sn, etc.). In turn, T*_x_* in the MXene formula represents the surface functional groups (such as −O, −F or −OH) introduced through the wet-chemical etching (see Figure 2) [57].

First, MXene was synthesized by Naguib et al. from Ti_3_AlC_2_ by the selective etching of Al with aqueous HF solution at room temperature. In this case, the synthesis process can be represented with the following equations [58]:

M*_n_*_+1_AX*_n_* + 3HF → AF_3_ + 3/2H_2_ + M*_n_*_+1_X*_n_*, (1)

M*_n_*_+1_X*_n_* + 3H_2_O → M*_n_*_+1_X*_n_*(OH)_2_ + H_2_, (2)

M*_n_*_+1_X*_n_* + 2HF → M*_n_*_+1_X*_n_*F_2_ + H_2_. (3)

**Figure 5:**
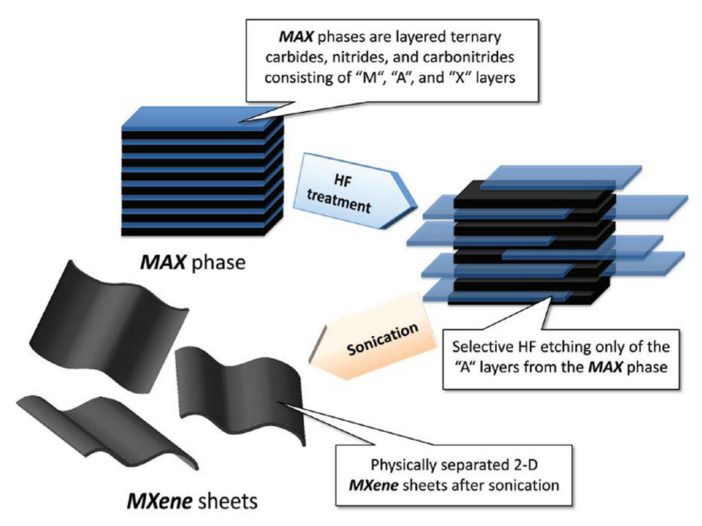
Formation of MXenes by etching and exfoliation of MAX phases. Reprinted with permission from [61]. Copyright © 2012 American Chemical Society.

The replacement of Al atoms by O, OH and F atoms leads to a decrease in the interaction of M*_n_*_+1_X*_n_* layers, allowing their separation and the formation of 2D “graphene-like” layers [59]. The typical synthesis process of MXene phases is illustrated in Figure 5 and includes several stages. First, MAX phases were treated with the etching solution, resulting in the formation of the terminated 2D layers bonded via hydrogen and van der Waals bonds [51]. In the next step, the cleaning procedures, including repetitive washing with DI water, centrifugation and filtration of MXene powders, were conducted to remove the residual acid and reaction products. Normally, this stage is performed until a pH of 6 is achieved. After the vacuum-assisted filtration followed by vacuum drying, the multilayered MXene structures can be obtained [57]. The production of the single-layered MXenes with unique functional properties requires their intercalation and delamination using sonication methods or by applying intercalating agents. Polar organic intercalants are considered the most effective for the MXenes’ delamination, as their use results in the weak interlayer interaction and increased interlayer distance [50]. Moreover, the hydrophilic nature of the MXene structures allows producing well-delaminated layers by their mild sonication or simple handshaking in water [60].

**Figure 6:**
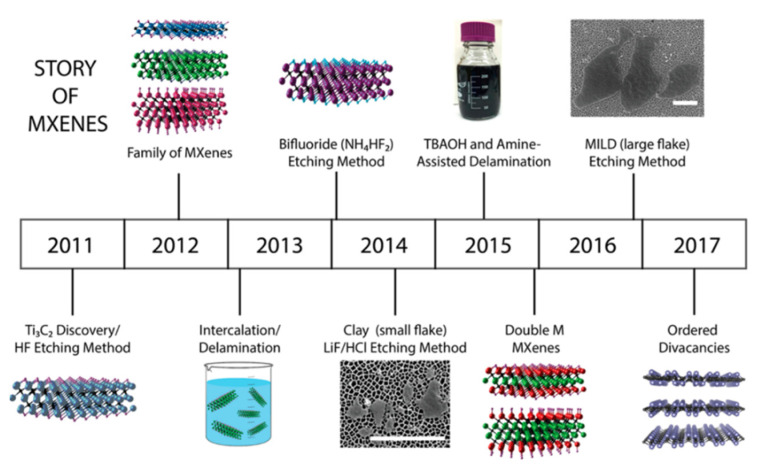
Stages of the MXenes development. Reprinted with permission from [57]. Copyright © 2017 American Chemical Society.

In their work, Alhabeb et al. have shown how the synthesis methods to obtain MXene structures have adjusted since their discovery in 2011 [57]. To date, MXene structures passed through several developing stages (see Figure 6), including the first synthesis of a multilayered MXene composition [51,61]; employment of intercalation/delamination techniques to obtain single-layered MXenes [62]; improvement of the etching process by using new etchants, such as ammonium bifluoride salt [63] and a LiF/HCl composition [64]; simplification through the shaking approach instead of sonication in the delamination stage (so-called MILD method—the minimally intensive layer delamination) [65].

All development stages allowed not only to improve the existing synthesis method but also to increase the diversity of the MXene family. As in the case of all nanomaterials, the morphology and, as a result, the properties and performance of the MXene structures in different applications can be controlled by adjusting the synthesis parameters, namely the concentration of the etching solution, etching and ultrasonic time and the process temperature. In turn, the quality and composition of MAX compounds, particle size, intercalating agent, type of the etching solution, as well as etching time and temperature cause significantly influence the etching procedure. For instance, a higher atomic number “M” requires the employment of more aggressive etching parameters, such as higher acid concentrations or longer etching time [50].

**Figure 7:**
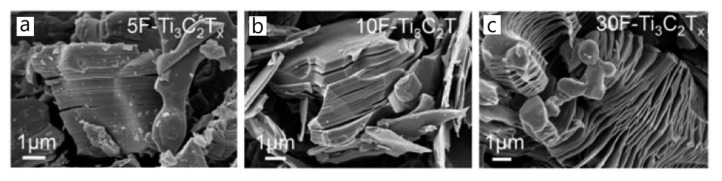
SEM images of the MXene structures synthesized using different etchant concentration: (**a**) 5 wt% HF; (**b**) 10 wt% HF; (**c**) 30 wt% HF. Reprinted with permission from [57]. Copyright © 2017 American Chemical Society.

Sang et al. showed that Ti_3_C_2_T*_x_* MXene could already be obtained using an etchant solution with 2.7 wt% HF concentration. Moreover, the authors confirmed that the increase in HF concentration leads to the formation of more defects in the MXene structures [65]. Conversely, low etchant concentration does not allow obtaining a well-isolated layered morphology. Alhabeb et al. studied the effect of HF concentration on the efficiency of Al elimination from the MAX phase, as well as on MXene morphology [57]. It was shown that a 5 wt% HF concentration is sufficient for etching aluminum selectively but demands a longer etching time in comparison to a higher HF concentration. The morphology of the obtained MXene structures is strongly affected by the etchant concentration, and an accordion-like morphology can be achieved only by the employment of a higher HF concentration of 30 wt% (see Figure 7). The authors explained that the formation of the accordion-like MXene morphology relies on the production of a large amount of H_2_ during the reaction with a higher concentration of HF with Al.

Thus, considering the influence of process parameters on the functional characteristics and performance of MXene structures in the desired application field, considerable attention should be paid to choosing the synthesis route. For instance, the slight adjustment of the HF-process results in the production of porous 2D MXenes with a larger specific surface area and more opened structures [66], which can provide better adsorption properties for gas sensing applications. The employment of the HCl/LiF etchant method not only increases the MXene yield and provides a safer process but also allows achieving the formation of clay-like materials, which can be easily shaped to obtain the desired forms for further applications, including sensing electrodes [64].

#### Alternative Non-Fluorine Safe Synthesis Routes

Since their discovery, more than 30 stoichiometric MXene structures have been synthesized, but the expanded research on MXene and their application in various fields requires additional investigations on improving the MXene quality and their functional performances [67]. In the case of sensing applications, synthesis not only controls the material’s morphology but also directly affects sensory functions, and therefore, new synthesis routes shall be explored and process parameters shall be adjusted. Considering the harmful HF effect on human health and the environment, non-hazardous HF-free etching methods deserve special attention due to their advantages, such as high exfoliation yield, low sonification time, fewer defects and easier handling [47].

One of the first non-HF etching methods was proposed by Ghidiu et al. with the employment of an HCl and LiF mixture, resulting in the HF generation during the process reaction. This method allowed higher MXene yields while providing a safer, easier and faster synthesis route [64]. Lipatov et al. developed a LiF-based etching method by adjusting the MAX phase and ratio of LiF. In their work, the authors fabricated high-quality Ti_3_C_2_T*_x_* MXene layers with a well-defined, defects-free structure, which showed stable performance and high conductivity [68].

Other safe, non-hazardous and non-toxic approaches for the MXene synthesis include the usage of the Lewis acidic molten salts (ZnCl_2_ [69], CuCl_2_ [70], etc.); alkali-assisted approaches (NaOH-assisted hydrothermal process [71]; NH_4_OH electrochemical etching [72]; halogens (Br_2_, I_2_, ICl, IBr) utilization in anhydrous media [73].

Besides the green chemistry aspect, such alternative non-fluorine etching methods allow increasing the number of MXene structures [70], as well as producing MXenes with new or improved characteristics. Thus, Li et al. applied a ZnCl_2_-based method in their work to synthesize Cl-terminated MXenes [69], which are expected to be more stable in comparison with F-terminated MXene structures and showed enhanced electrochemical characteristics [69,70]. Jawaid et al. proposed the efficient room-temperature etching method using halogens and showed that the suggested method provides opportunities for the controlled surface chemistry of MXenes, and as a result, the modulation of the MXene properties [73]. Moreover, the absence of dangerous agents in the production process makes non-fluorine etching methods appealing for industrial MXene materials production [72].

Despite the tremendous progress made in MXene synthesis routes, wet etching synthesis approaches remained the main methods for MXene production. However, recently, other processing techniques were suggested to obtain MXene structures. These include the chemical vapor deposition method [74] and template synthesis [75,76]. For instance, Xu et al. demonstrated the suitability of the CVD method for obtaining high-quality Mo_2_C nanosheets with a large lateral size of ~10 μm and a few nanometers thick. Moreover, the proposed CVD process allows obtaining ultrathin WC and TaC crystals, enabling the expansion of the 2D materials family [77].

### 3.2. High-Performance MXene-Based Gas Sensors

Owing to the 2D layered graphene-like morphology, MXene structures are ideal materials for building high-performance sensors, considering their adjustable surface terminations and unique surface chemistry, metallic conductivity, tunable bandgap, easy functionalization and excellent mechanical strength [24,46,47]. To date, successful applications of MXenes in gas sensors, strain/stress sensors, electrochemical and optical detectors and humidity sensors [24] have been published.

#### 3.2.1. MXenes’ Sensing Mechanism

It can be immediately noticed that the sensing mechanism in the MXene structures differs from that of the metal oxides and is more complicated than the surface adsorption or charge transfer in conventional 2D materials [52]. It is well known that the sensing mechanism of sensors based on metal oxides depends on the surface reactions of gas molecules with pre-adsorbed oxygen species [78]. The fabrication of composites or hybrid structures leads to changes in the sensing mechanism, while added compounds act as a “catalyst” for the improvement of sensing properties of the base sensing material [8].

**Figure 8:**
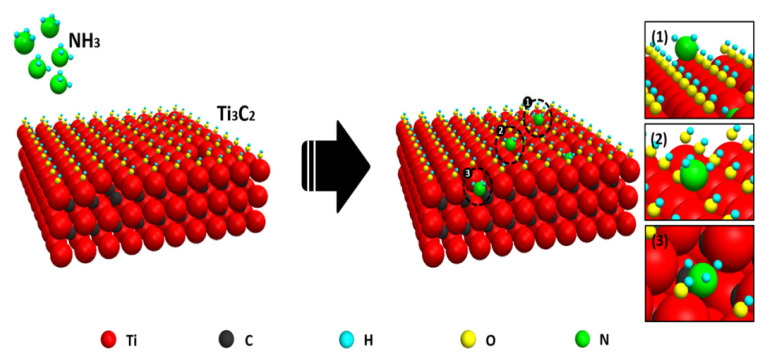
Schematic illustration of the possible MXene gas sensing mechanism. Reprinted with permission from [7]. Copyright © 2017, American Chemical Society.

The sensing mechanism of the MXene materials depends on the charge transfer process, which is based on the physisorption of gas molecules on the surface without involving the adsorbed oxygen species [8]. Thus, the change in the electrical properties is caused by the adsorption/desorption process. Figure 8 shows the schematic illustration of the possible gas sensing mechanism in the Ti_3_C_2_ MXene compound. For example, in the case of ammonia adsorption on the Ti_3_C_2_T*_x_* surface, the resistance increase occurs due to the combination of generated electrons with holes in MXene [24]:

2NH_3_ + 3O^−^ → N_2_ + 3H_2_O + 3e^−^, (4)

NH_3_ + OH^−^ → NH_2_ + H_2_O + e^−^. (5)

**Figure 9:**
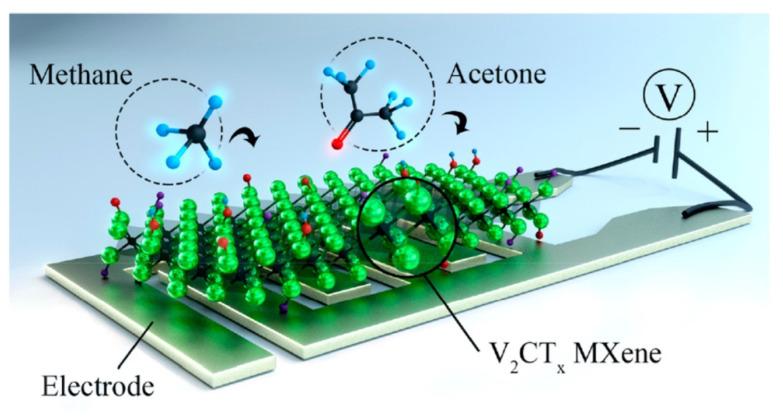
Schematic illustration of the possible sensing mechanism suggested for the V_2_CT*_x_* MXene based gas sensor. Reprinted with permission from [49]. Copyright © 2017, American Chemical Society.

Lee et al. proposed a possible sensing mechanism for the V_2_CT*_x_*-based sensor (see Figure 9), suggesting that oxygen-terminated groups significantly contribute to the receptor function similarly to the Ti_3_C_2_T*_x_* MXene. The authors also concluded that hydrophilic groups are preferred for the gas species adsorption compared to the hydrophobic and fluorine groups [49].

Ti_3_C_2_T*_x_*-based sensitive layers show the increasing resistivity for the detection of both oxidizing and reducing gases, and due to the metallic-like conductivity, the complication of charge transfer occurs with gas adsorption [23]. When detecting such gases, such as ethanol, methanol, acetone or ammonia, Ti_3_C_2_T*_x_* MXene shows p-type semiconducting properties, which is probably attributed to the adsorbed water molecules introduced during the Al etching process [7]. Lee et al. assumed that the sensing mechanism of the MXene structures involves the gas adsorption both by structural defects and presented functional groups. The replacement of the surface functional groups by gas molecules leads to the carrier transfer between adsorbent and adsorbed gases, resulting in the significant change of MXene resistance value [7].

#### 3.2.2. Sensors Based on Pure MXenes

**Figure 10:**
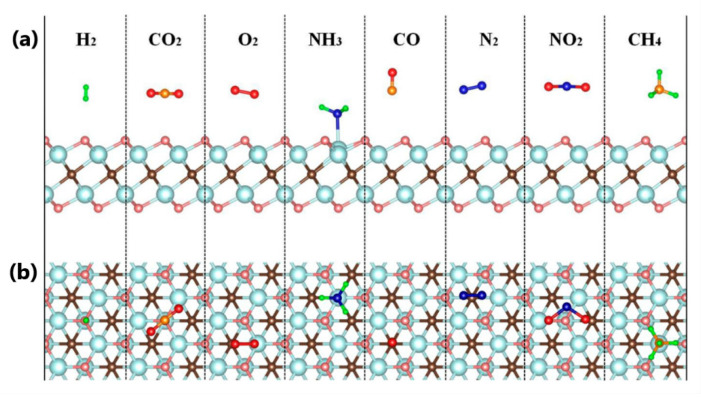
Schematic illustration of the gas adsorption on the Ti_2_CO_2_ monolayer: (**a**) side view; (**b**) top view. Reprinted with permission from [79]. Copyright © 2015, American Chemical Society.

The potential of MXene compounds for gas sensor applications was firstly demonstrated by Yu et al. using theoretical simulation [79]. Density functional theory (DFT) was applied to investigate the electronic structure of Ti_2_CO_2_ monolayer for NH_3_ detection. By using the first-principle simulation, the authors show high sensitivity and selectivity of the Ti_2_CO_2_ layer to NH_3_. Moreover, the obtained value of NH_3_ adsorption energy confirmed that the Ti_2_CO_2_ sensor could recover easily after gas detection (see Figure 10).

Similar results using the DFT method were obtained by Xiao et al. for M_2_CO_2_ MXenes (M = Sc, Ti, Zr, Hf). In addition, the authors concluded that the efficient release and capture of NH_3_ gas on the MXene surface could be controlled due to the variation of the M_2_CO_2_ charge state [80].

As mentioned above, the perspectives of MXenes application to the chemoresistive gas sensors are due to the large specific surface area, high conductivity, surface functionality and hydrophilicity [21]. The hydrophilic characteristic is introduced during the etching process of MXene structures and depends strongly on the surface terminations (−OH, −O, −F, −Cl terminal groups). As a result, MXenes may be ideal sensing candidates due to the adsorption of polar (hydrophilic) gas molecules but may have limited functionality in the detection of polar (hydrophobic) molecules [51].

**Figure 11:**
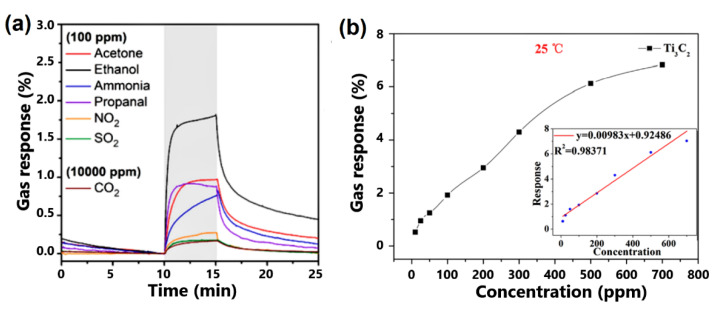
(**a**) Gas sensor response of Ti_3_C_2_T*_x_* MXene to different gases at the concentration of 100 ppm at room temperature. Reprinted with permission from [6]. Copyright © 2018, American Chemical Society. (**b**) Gas sensor response of Ti_3_C_2_ MXene to different NH_3_ concentrations at room temperature. Reprinted with permission from [81]. Copyright © 2019, American Chemical Society.

The further investigation of MXene-based gas sensor devices proved their sensing performance. Thus, the study of sensing performance of the Ti_3_C_2_T*_x_*, obtained using LiF/HCl etching method, showed good responses of Ti_3_C_2_T*_x_*-based sensor under exposure to several gases with the concentration of 100 ppm (see Figure 11a) [6]. The obtained results also confirmed the difference in the sensing mechanism of MXenes in comparison to the semiconducting materials. In contradiction to the semiconductor gas sensors, for which the response depends strongly on the electron donor/acceptor properties and the type of charge carrier, the resistance of MXenes under gas exposure always increases and is not affected by the gas type (oxidizing or reducing). The last is connected with the complication of charge carrier transport with gas adsorption. This additional sensing property is of importance for simultaneous, selective sensing of NO, NO_2_ and CO, CO_2_ in mixed gas environments.

Wu et al. applied the NaF/HCl etching approach with subsequent delamination with dimethyl sulfoxide to obtain single-layered Ti_3_C_2_ MXene [81]. Synthesized structures were tested for 500 ppm concentration of different gases, including methane, H_2_S, ethanol, methanol, acetone and ammonia. Among all tested gases, MXene structures showed higher sensitivity and selectivity to NH_3_ with an almost linear sensor response in the concentration range of 10–700 ppm (see Figure 11b). Furthermore, the authors concluded that the etchant has a direct effect on the selectivity of NH_3_. Thus, the selectivity of Ti_3_C_2_ synthesized using the NaF/HCl etching solution is significantly higher compared to the selectivity of MXene structures obtained using the LiF/HCl etching method. This is probably connected with the higher number of the adsorption centers due to the easier removal of Na ions from the Ti_3_C_2_ surface in comparison with that of Li^+^.

**Figure 12:**
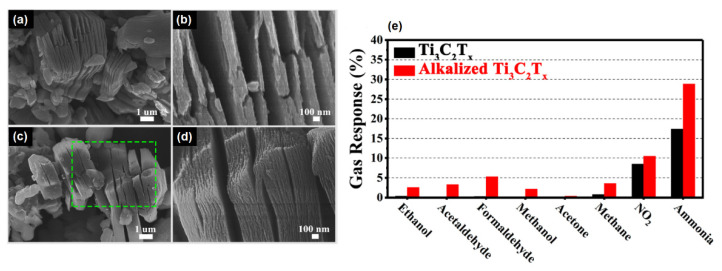
Morphology and sensor response of Ti_3_C_2_T*_x_* and alkalized Ti_3_C_2_T*_x_*: (**a**,**b**) SEM images of Ti_3_C_2_T*_x_*; (**c**,**d**) SEM images of alkalized Ti_3_C_2_T*_x_*; (**e**) gas sensor response of Ti_3_C_2_T*_x_* and alkalized Ti_3_C_2_T*_x_* to the test gases with a concentration of 100 ppm. Reprinted with permission from [82]. Copyright © 2019, American Chemical Society.

Besides the synthesis method and altering the etching parameters, the functionalization of MXene surfaces significantly contributes to the gas sensor performance. For instance, it was possible to improve the MXene gas sensing properties by alkaline treatment [82]. In their work, Yang et al. synthesized Ti_3_C_2_T*_x_* using the HF-etching method with the following NaOH treatment to obtain an alkalized MXene structure. Figure 12a–d shows the morphological characteristics of both Ti_3_C_2_T*_x_* and alkalized Ti_3_C_2_T*_x_*. The obtained structures were tested with humidity and several different gases with a concentration of 100 ppm. Sensor devices based on the alkalized Ti_3_C_2_T*_x_* structures showed considerably better sensor performance. For instance, sensor response to NH_3_ was 29% and 17% for alkalized and non-alkalized Ti_3_C_2_T*_x_*, correspondingly (see Figure 12e). The authors explained the enhanced sensing characteristics by Na^+^ intercalation and increment of O^−^ terminals.

**Figure 13:**
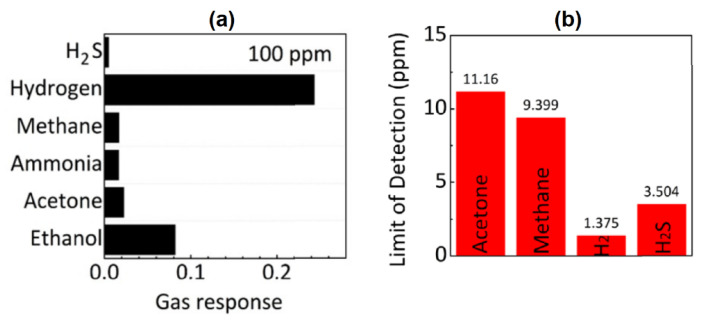
(**a**) Gas sensor response of V_2_CT*_x_* to the various gases with a concentration of 100 ppm at room temperature. (**b**) The theoretical detection limit of the V_2_CT*_x_*-based sensor to various gases. Adapted with permission from [49]. Copyright © 2019, American Chemical Society.

To date, Ti_3_C_2_T*_x_* structures are the most explored ones for gas sensor applications. However, recently, Lee et al. demonstrated the perspective of vanadium carbide for the fabrication of ultrahigh sensitive gas sensors [49]. The developed V_2_CT*_x_*-based sensors showed a sensor response at room temperature for both polar (H_2_S, ammonia, acetone, ethanol) and non-polar gases (H_2_ and Methane) with a concentration of 100 ppm (see Figure 13a). Furthermore, the calculated theoretical detection limit for hydrogen and methane of 1 ppm and 9 ppm, respectively, were obtained (see Figure 13b), which is significantly lower than the values reported before.

Zhao et al. showed new perspectives of V_3_C_2_T-based sensors for medical applications, namely for the earlier diabetes diagnosis by detection of the trace acetone concentrations. The developed sensor showed a detection limit that is lower than the diabetes diagnosis threshold: 1 ppm in comparison with 1.8 ppm [83].

Table 1 presents the data and references on sensors based on pure MXene structures.

#### 3.2.3. MXenes-Based Heterostructures as Sensitive Layers

Besides varying the morphology, the formation of the MXene-based heterostructures and composites is an effective way of improving their gas sensor performance. To date, several works were conducted on the synthesis of MXene-based composites using the hydrothermal route [86], wet spinning method [24,87], self-assembly process [48] and spray pyrolysis [88] synthesis approaches. The study of gas sensing properties of the obtained compounds showed great perspectives for their applications in this field.

One of the most often used methods for the improvement of MXene sensor properties is their modification with metal oxides. For instance, Tai et al. demonstrated the enhanced sensor performance of Ti_3_C_2_T*_x_* nanosheets modified with TiO_2_ nanoparticles. Thus, a gas sensor based on the TiO_2_/Ti_3_C_2_T*_x_* composite showed a 1.6 times higher response and 0.65/0.52 shorter response time to 10 ppm NH_3_ in comparison to pure MXene [89]. In situ growth of TiO_2_ nanowires on the Ti_3_C_2_ surface allows obtaining a composite with a significantly increased surface area compared to pure Ti_3_C_2_ or TiO_2_ materials, resulting in the production of a highly sensitive humidity sensor [90].

He et al. synthesized MXene structures decorated with SnO_2_ nanoparticles, which exhibit excellent sensor performance to the NH_3_ with the detection limit at 0.5 ppm at room temperature response/recover time shorter than 30 s [86]. The authors explained that the superior sensing properties of the fabricated structures by the increased electron numbers on the SnO_2_ surface are due to the difference in the Fermi level of the MXene and SnO_2_. Moreover, MXene structures provide a unique matrix with selective adsorption abilities.

**Figure 14:**
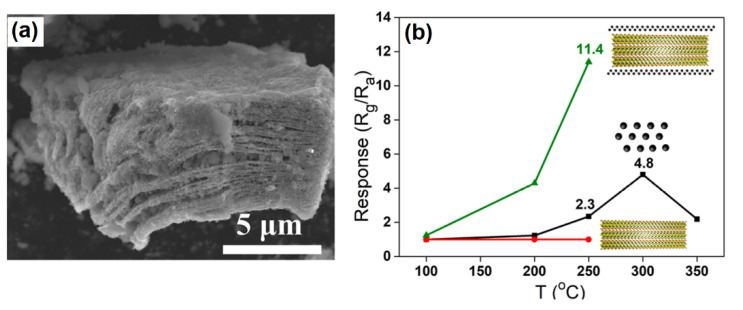
(**a**) TEM image of T_3_C_2_T*_x_*/CuO composite. (**b**) Gas sensor response of CuO, T_3_C_2_T*_x_* and T_3_C_2_T*_x_*/CuO composite to 50 ppm of toluene. Adapted with permission from [48]. Copyright © 2020, American Chemical Society.

Herwaman et al. prepared hybrid heterostructures of CuO nanoparticles and Ti_3_C_2_T*_x_* MXenes using the electrostatic self-assembly approach and demonstrated that CuO/Ti_3_C_2_T*_x_* composite exhibits 5 times higher sensor response to toluene in comparison with pristine CuO (see Figure 14). Moreover, the authors achieved improved selectivity and response/recovery times due to the high MXene phase metallic conductivity [48].

Besides using metal oxides, the investigation of the enhanced sensing performance of MXene’s by their modification with metals and the fabrication of MXene/carbon materials composites was conducted.

**Figure 15:**
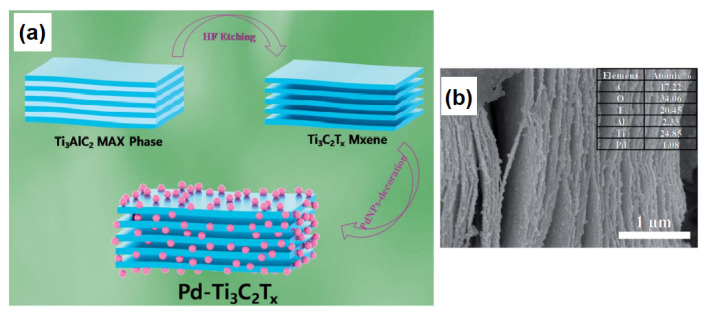
(**a**) Schematic illustration of Pd-Ti_3_C_2_T*_x_* formation. (**b**) SEM image of the Pd-decorated Ti_3_C_2_T*_x_*. Republished with permission from [91]. Royal Society of Chemistry, Creative Commons Attribution 3.0 Unported License.

For instance, the decoration of MXene surfaces with Pd leads to the enhancement of the gas sensor’s response to H_2_ at room temperature [91,92]. Doan et al. [91] have grown Pd nanoparticles on the Ti_3_C_2_T*_x_* using a polyol method (see Figure 15) and demonstrated that the adjustment of the Pd concentration and Pd-particle distribution on the MXene’s surface has an influence on the formation of high-performance hydrogen sensors that exhibited sharp, concentration-dependent and selective responses even at room temperatures.

**Figure 16:**
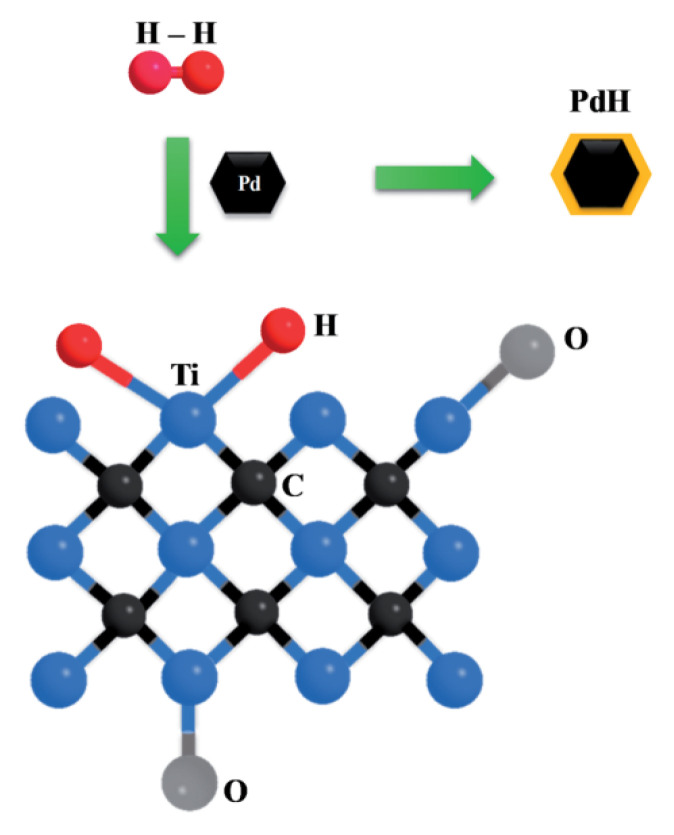
Schematic illustration of H_2_ sensing mechanism of the Pd-decorated Ti_3_C_2_T*_x_*. Republished with permission from [91]. Royal Society of Chemistry, Creative Commons Attribution 3.0 Unported License.

Moreover, Doan et al. explained that the superior H_2_ sensing capability of the obtained MXene/Pd structures depends on the formation of PdH due to the adsorption and dissociation of H_2_ molecules promoted through the Pd catalytic nature (see Figure 16) [91].

**Figure 17:**
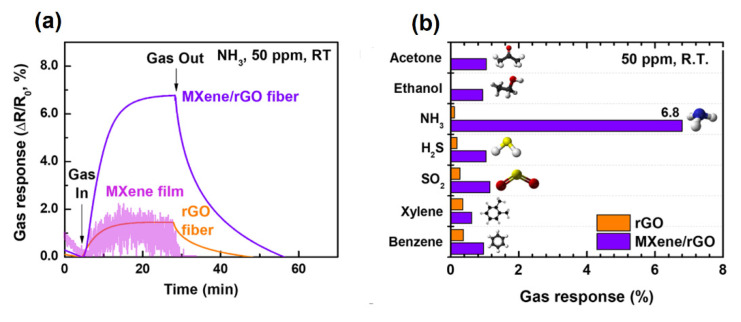
(**a**) Gas sensor response of MXene film, rGO fiber and MXene/rGO hybrid fiber to 50 ppm NH_3_ at room temperature. (**b**) Gas sensor response of rGO fiber and MXene/rGO hybrid fiber to various testing gases at concentrations of 50 ppm. Republished with permission from [87]. Copyright © 2020, American Chemical Society.

The fabrication of MXene/GO fibers allows improving the sensor’s response compared to other individual materials due to the MXene/graphene synergetic effect of electronic and adsorption properties [87]. The obtained hybrids exhibit an improved sensing response to NH_3_ at room temperature (see Figure 17), providing high mechanical flexibility, which makes them a potential material for wearable gas sensor devices.

**Figure 18:**
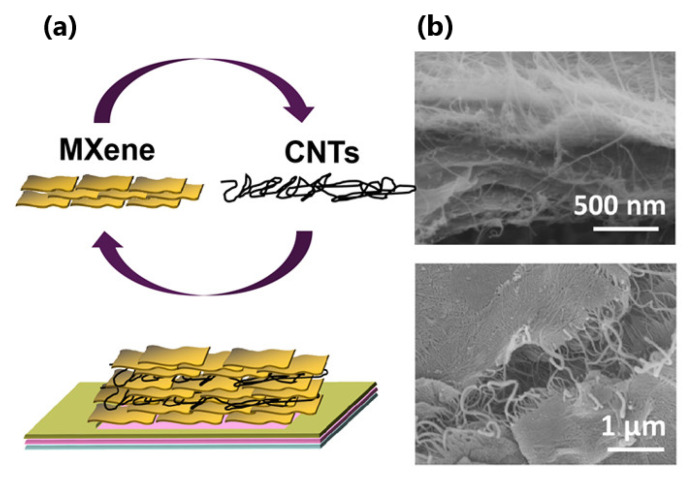
(**a**) Fabrication process of the Ti_3_C_2_T*_x_* MXene/CNT layer. (**b**) SEM images of the Ti_3_C_2_T*_x_* MXene/CNT composite [94]. Copyright © 2018, American Chemical Society.

The incorporation of the carbon nanotubes in the MXenes structures allows preventing the restacking of the Mxene nanosheets during the fabrication process (see Figure 18), resulting in the enlargement of specific surface area and pore volume [93].

**Figure 19:**
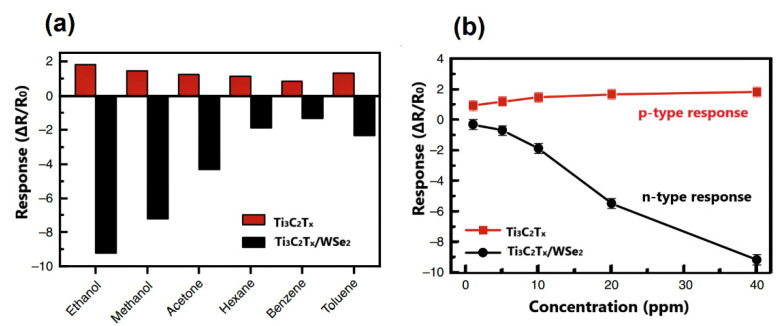
(**a**) Gas sensor response of Ti_3_C_2_T*_x_* and Ti_3_C_2_T*_x_*/WSe_2_ sensors to different gases with a concentration of 40 ppm. (**b**) Gas sensor response of Ti_3_C_2_T*_x_* and Ti_3_C_2_T*_x_*/WSe_2_ sensors to ethanol Republished with permission from [55]. Copyright © 2020, Springer Nature, Creative Commons Attribution 4.0 International License.

In their work, Cai et al. showed perspectives of MXene/CNT composites for the development of ultrahigh sensitive strain sensors due to the combination of good MXene electric properties and excellent conductivity and stretching ability of the carbon nanotubes [94]. On the other hand, MXene/CNT hydride structures can be promising for the development of high-performance gas sensor devices on their basis. For this purpose, novel approaches for the CNTs growing and fabrication of MXene/CNT composite materials may be considered [95,96]. Chen et al. demonstrated the enhancement of MXenes sensing properties for the detection of oxygen-containing volatile organic compounds (VOCs) by their hybridization with transition metal dichalcogenides [55]. Ti_3_C_2_T*_x_*/WSe_2_ nanohybrid composites, fabricated using surface-treating and exfoliation-based process, were tested for the detection of several VOCs and showed significant improvement of sensor response to the ethanol, methanol and acetone (see Figure 19a). Thus, the sensitivity of Ti_3_C_2_T*_x_*/WSe_2_ nanohybrids to ethanol was increased by over 12-fold compared to the pristine Ti_3_C_2_T*_x_* MXene, showing an almost linear response at different ethanol concentrations (see Figure 19b). Interestingly, the formation of Ti_3_C_2_T*_x_*/WSe_2_ composite led to the change in the type of sensing behavior from p- to n-type.

**Figure 20:**
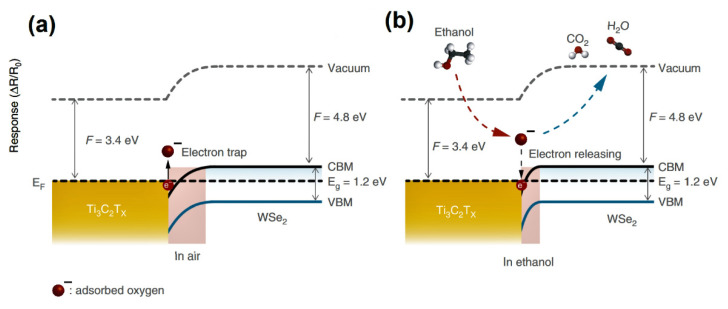
Energy-band diagram of the Ti_3_C_2_T*_x_*/WSe_2_ in (**a**) air and (**b**) ethanol. Republished with permission from [55]. Copyright © 2020, Springer Nature, Creative Commons Attribution 4.0 International License.

Based on the obtained results authors proposed the sensing mechanism for the Ti_3_C_2_T*_x_*/WSe_2_ nanohybrids, explaining the enhancement of sensing reactions due to the flow of electrons from highly conductive MXene to WS_2_ surface (see Figure 20). At ambient, the depletion layer is forming due to the trapping of electrons (see Figure 20a). Under the gas exposure (for instance, ethanol), the release of electrons back to the conduction band is occurring, resulting in the decrease in the depletion layer and a corresponding decrease in sensor resistance (see Figure 20b).

Conductive polymers are the other very promising class of materials for gas sensing applications, considering the number of their advantages. In comparison with metal oxide-based sensor devices, they exhibit high sensitivity and short response time at room temperatures, are characterized by good mechanical properties and are easy for manufacturing [97]. To a date such conductive polymers, as polyaniline (Pani) [98,99], polythiophene (PTh) [100], polypyrrole (PPy) [101,102,103], poly(3,4-ethylenedioxythiophene) (PEDOT) [104] and polyacetylene (PA) [105] were used for the gas sensor fabrications, which showed good respond to the variety of gasses, including NH_3_ [106,107], NO_2_ [108,109], H_2_S [110,111], Ethanol [112], Methanol [113], etc.

Among a list of potential polymers, PANI is the most widely used in sensors [114], for instance, as an NH_3_ sensor, because the amine groups have excellent gas sensitivity to the nitrogen-containing substances [115]. Conversely, MXenes contain a large number of oxygen-terminated groups, which can strongly interact with NH_3_, resulting in an excellent gas sensor response [86]. The formation of Mxene/PANI composites leads to superior sensing properties not only to the NH_3_ but other gas substances as well. Thus, in their work, Wang et al. achieved an improved NH_3_-sensing response in a high-humidity environment by the development of Nb_2_CT*_x_* nanosheets/PANI nanofibers composites. The sensor device based on the obtained composite showed a good linear response to NH_3_ in the concentration range of 1–100 ppm at room temperature. The authors assumed that the reduced influence of humidity could be the result of occupied active sites for water adsorption due to the formation of intermolecular Nb_2_CT*_x_*/PANI hydrogen bonds [116].

Zhao et al. fabricated the Ti_3_C_2_T*_x_* nanosheets decorated with PANI nanoparticles and demonstrated that the developed composites are characterized by remarkable sensitivity to ethanol at room temperature, providing fast response/recovery times and good mechanical stability [117]. The authors explained that the improved sensor performance was due to the synergetic properties of the nanocomposite, namely by the increased number of gas adsorption sites due to the large surface areas of composite material and large number of functional groups on the MXene’s surface.

PEDOT:PSS is another studied polymer for the fabrication of MXene/polymer-based gas sensors due to its simple production and high conductivity [22]. The combination of PEDOT:PSS with Ti_3_C_2_T*_x_* results in a synergetic effect with enhanced sensitivity to NH_3_ in comparison to both pure PEDOT:PSS- and Ti_3_C_2_T*_x_* MXene-based sensors due to the large active sites on the MXene’s surface and direct charge transfer in PEDOT:PSS/Ti_3_C_2_T*_x_* composite structure [118]. Table 2 presents the information on sensors based on MXene composites and heterostructures.

#### 3.2.4. Two-Dimensional MXene Layered Materials for Gas Sensor Application

Thus, considering the set of their functional properties, MXene materials are potential candidates for sensing applications. However, the stacking of Ti_3_C_2_T*_x_* nanosheets together can lead to a decrease in specific surface areas, resulting in their limited sensing performance [122]. It is well known that for gas sensing applications, 2D layered structures deserve special attention due to their numerous advantages, including a large surface-to-volume ratio, excellent flexibility, tunable electronic structure and excellent mechanical stability [24].

**Figure 21:**
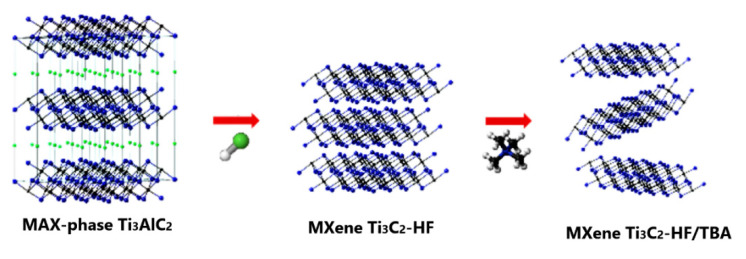
Schematic illustration of the Ti_3_AlC_2_ exfoliation. Reprinted with permission from [123]. Copyright © 2020, American Chemical Society.

The fabrication of MXene structures from MAX phases by etching process typically leads to the formation of multilayered structures. As was shown above, the morphology of the MXene structures can be adjusted by varying process conditions, namely the increase in HF concentration leads to the formation of good isolated accordion-like MXene layers. To obtain single-layered MXene structures, an additional exfoliation step is required (see Figure 21), usually followed by sonication or mechanical shaking [60]. The intercalation or delamination allows the discovery of unique properties of 2D materials.

The first f-Ti_2_C_2_ MXene intercalation was achieved by Mashtalir et al. by inserting organic molecules into the interlayers of accordion-like MXene structures. In their work, the authors showed that the intercalation of dimethyl sulphoxide enabled the delamination of the stacked f-Ti_3_C_2_ layers into separate 2D MXene sheets. Furthermore, the authors confirmed that intercalated samples were characterized by electrical resistivity compared to the non-intercalated MXenes [62].

The choice of the intercalant, as well as process parameters, has a significant effect on the material’s functional and sensing properties. To date, several intercalants were used for the MXene exfoliation into single layers, including tetrabutylammonium hydroxide [60,124], hydrazine [62,125], dimethyl sulfoxide [62,126], urea [62,126] and isopropyl amine [126]. For instance, Mashtalir et al. investigated the effect of the hydrazine intercalation on the structure and properties of Ti_3_C_2_-based MXene and concluded that hydrazine intercalation results in surface chemistry changes by decreasing the number of fluorine and OH surface groups. Moreover, the opening of more active sites on the MXene surface occurs [125], leading to better adsorption and sensing properties of the material.

Chia et al. showed that the exfoliation method has a strong influence on the sensing performance. Thus, Ti_3_C_2_ structures, obtained via HF-etching and subsequent delamination with tetrabutylammonium hydroxide, exhibited high selectivity and excellent electrocatalytic activity in the proposed biosensing system for glucose detection, opening the way for the MXene applications in biomedical and food sampling areas [123].

**Figure 22:**
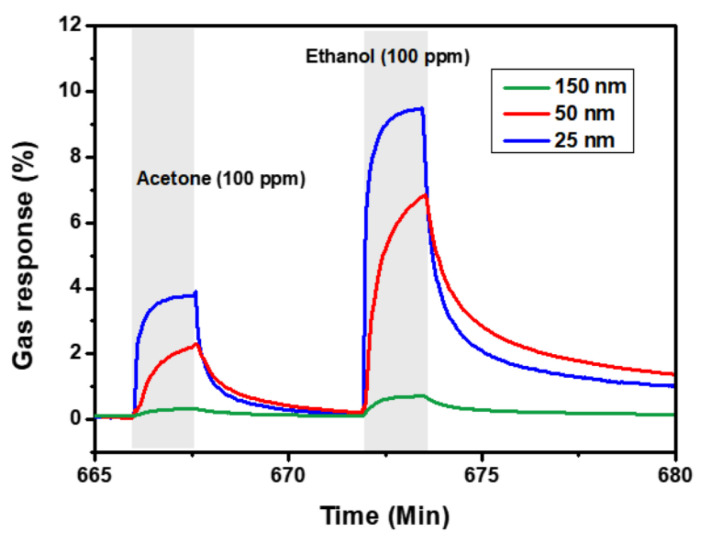
Gas sensor response of Ti_3_C_2_T*_x_* MXene with different thicknesses to acetone and ethanol at the concentration of 100 ppm. Reprinted with permission from [6]. Copyright © 2018, American Chemical Society.

The other important parameter of the layered MXene structures for gas sensor applications is the layers’ thickness (see Figure 22). Thus, thinner MXene films exhibit a higher sensor response, which is apparently connected with active sites on the exposed MXene structure, where −OH groups play an important role in the gas species adsorption [6].

## 4. Conclusions

This review describes the most recent theoretical (using density functional theory simulations) and experimental studies related to MXene-based compounds, especially for gas sensing applications. Various synthesis approaches of the literature are introduced detailing the production of Mxene, the development of pure Mxene compounds as well as Mxene containing hybrid composite nanostructures. The literature indicates that the synthesis method controls the material’s morphology. For instance, a varying etchant concentration led to the formation of the well-isolated accordion-like structures that provide a larger specific surface area and better adsorption properties. Additionally, these properties are preferable for the development of high-performance sensing devices. In order to eliminate the harmful effects of HF-based synthesis, the literature paid particular attention to the employment of less hazardous, HF-free and greener etching methods. These are mainly the usage of the Lewis acidic molten salts and alkali-assisted approaches.

Giving more emphasis to the gas sensors, the sensing mechanism of the developed MXenes has been introduced, which differs from that of the semiconducting metal oxides. It has been shown that the sensing mechanism in MXene structures follows a more complicated route and depends on the charge transfer process based on the physisorption of gas molecules on the surface without involving the adsorbed oxygen species. Gas sensor applications employ pure MXenes and their composites, although the sensing mechanism of MXene-based hybrid structures exhibits differences. Literature data indicate that the formation of MXene composite materials increases the specific surface area, which leads to the bigger interlayer spacing and introduction of new active sites for gas adsorption.

Since the first discovery of MXene compounds, significant progress in their synthesis and structural design has been achieved that expanded the MXene family. However, considering the ongoing demand for higher performance materials for different applications, these studies can still be considered in their early stages. For instance, the controllable synthesis of MXene compounds with a particular size, defects and surface terminations, including monolayered structures, remains a challenge. The literature data shows that there is a great perspective of MXene hybrid composites and heterostructures for gas sensing applications; however, further and extensive investigation of their combinations with other elements, compounds and stacking structures is needed.

The available, up-to-date research is devoted to the development of MXene-based sensing devices, especially for room temperature applications. These display some thermal instability problems. Therefore, future development should pay particular attention to the improvement of their chemical and thermal stability, especially at high temperatures and in humid environments, as well selectivity and sensitivity of the sensors. Moreover, additional studies are necessary to be conducted by the employment of other sensor designs (e.g., avoid the usage of more costly interdigital electrode structures).

## Figures and Tables

**Table 1:** List of the selected sensors prepared using pure MXene structures.

MXene	Preparation Method	Test Gas	Concentration (ppm)	Detection Limit	Sensitivity (Rg−Ra)/Ra	Response/Recovery Time	Ref.
Ti_3_C_2_T*_x_*	LiF/HCl-etching	Ethanol	100	100 ppb	1.7	-/-	[6]
		Acetone		50 ppb	0.97	-/-	
		Ammonia		100 ppb	0.8	-/-	
Ti_3_C_2_T*_x_*	LiF/HCl-etching	Methanol	100	-	0.143	-/-	[7]
		Ethanol		-	0.115	-/-	
		Acetone		25 ppm	0.075	-/-	
		Ammonia		-	0.21	-/-	
V_2_CT*_x_*	HF-etching	Acetone	100	11.16 ppm	0.0226	-/-	[49]
		Methane		9.39 ppm	0.0167	8/5 min	
		H_2_		1.375 ppm	0.2435	2/7 min	
		H_2_S		3.504 ppm	0.005	-/-	
Ti_3_C_2_	NaF/HCl-etching	Ammonia	500	10 ppm	6.13%	~2 min/ ~4 min	[81]
Ti_3_C_2_T*_x_*	HF-etching	NO_2_	100	-	8%	-/-	[82]
		NH_3_	100	-	17%	-/-	
	HF-etching + Alkalization	NO_2_	100	-	11%	-/-	
		NH_3_	100	10 ppm	29%	-/-	
V_4_C_3_T*_x_*	HF-etching	Acetone	100	1 ppm	2.5	-/-	[83]
Ti_3_C_2_T*_x_*	Electrospinning technique	Acetone	ppb level	50 ppb	1.4%	<2 min	[84]
		Ethanol			1.75%		
		Methanol			2.2%		
Mo_2_CT*_x_*	HF-etching	Toluene	100	-	2.65%	-/-	[85]

**Table 2:** List of the selected sensors based on MXene composite structures.

MXene	Synthesis Method	Test Gas	Concentration (ppm)	Detection Limit (ppm)	Sensitivity, (Rg−Ra)/Ra	Response/Recovery Time	Ref.
Ti_3_C_2_T*_x_*/CuO	electrostatic self-assembly	Toluene	50	-	11.4% *	270 s/10 s	[48]
Ti_3_C_2_T*_x_*/WSe_2_	Surface treating and exfoliation-based process	Ethanol	40	1	12%	9.7 s/6,6 s	[55]
Ti_3_C_2_T*_x_*/rGO	wet spinning	NH_3_	100	10	7.2%	-/-	[87]
Ti_3_C_2_T*_x_*/ZnO	spray pyrolysis	NO_2_	100	-	41.9%	34 s/105 s	[88]
Ti_3_C_2_/TiO_2_	hydrothermal	Humidity	7–97%	-	1614 pF/%RF	2.0 s/0,5 s	[90]
Ti_3_C_2_T*_x_*/Pd	polyol	H_2_	100	-	56%	-/-	[91]
Ti_3_C_2_T*_x_*/Pd	all-colloidal solution-based vacuum-filtration process	H_2_	4000	-	23%	37 s/161 s	[92]
Nb_2_CT*_x_*/PANI		NH_3_	100	-	301.31%	105 s/143 s	[116]
Ti_3_C_2_T*_x_*/PANI	wet chemistry	Ethanol	200	-	41.1%	0.4 s/0.5 s	[117]
Ti_3_C_2_T*_x_*/SnO-SnO_2_	hydrothermal	Acetone	100	-	12.1% *	18 s/9 s	[119]
Ti_3_C_2_T*_x_*/ Fe_2_(MoO_4_)_3_	hydrothermal	VOCs	5−1000	5	43.1%	18/24 s	[120]
Ti_3_C_2_T*_x_*/PANI	self-assembly	NH_3_	50	-	400%	-/-	[121]

* S = (Rg/Ra).
